# Towards preferential selection in the prisoner’s dilemma game

**DOI:** 10.1371/journal.pone.0282258

**Published:** 2023-02-24

**Authors:** Bingzhuang Qiang, Lan Zhang, Changwei Huang

**Affiliations:** 1 School of Computer, Electronics and Information, Guangxi University, Nanning, Guangxi, China; 2 School of Information, Xi’an University of Finance and Economics, Xi’an, Shanxi, China; 3 Guangxi Key Laboratory of Multimedia Communications and Network Technology, Guangxi University, Nanning, Guangxi, China; University of Electronic Science and Technology of China, CHINA

## Abstract

In previous works, the choice of learning neighbor for an individual has generally obeyed pure random selection or preferential selection rules. In this paper, we introduce a tunable parameter *ε* to characterize the strength of preferential selection and focus on the transition towards preferential selection in the spatial evolutionary game by controlling *ε* to guide the system from pure random selection to preferential selection. Our simulation results reveal that the introduction of preferential selection can hugely alleviate social dilemmas and enhance network reciprocity. A larger *ε* leads to a higher critical threshold of the temptation *b* for the extinction of cooperators. Moreover, we provide some intuitive explanations for the above results from the perspective of strategy transition and cooperative clusters. Finally, we examine the robustness of the results for noise *K* and different topologies, find that qualitative features of the results are unchanged.

## Introduction

Cooperation is ubiquitous in nature and human society [1]. However, this is inconsistent with Darwin’s theory of natural selection, because selfish individuals often give up cooperation in pursuit of higher payoffs. Therefore, the evolution and maintenance of cooperation is an interesting and attractive topic that has received widespread attention. Evolutionary game theory provides a powerful framework for understanding and unraveling the essence of the emergence of cooperation among unrelated individuals [2]. As a classical paradigm in game theory, the prisoner’s dilemma game (PDG) has been widely studied [3, 4]. The PDG describes the interaction between two individuals, with both individuals being allowed to cooperate with or betray their opponent. The individual’s payoff depends on the strategy that they choose. Mutual cooperation is rewarded *R*, and mutual defection is punished *P*. When a defector meets a cooperator, the defector will face temptation *T*, and the cooperator receives *S* (the sucker’s payoff). These payoffs need to satisfy *T* > *R* > *P* > *S* and *T* + *S* < 2*R*. Compared with cooperation, the defection is the optimal option. But they will obtain the maximal payoff when both players choose to cooperate. Therefore, a social dilemma is formed.

In the past few decades, researchers have put a great deal of effort into understanding how cooperative behavior can evolve in social dilemmas [5–7]. In a pioneering work, Nowak and May [8] first introduced a spatial structure into the PDG and found that cooperators can form clusters to resist the invasion of defectors. In addition, many specific mechanisms have been proposed to solve the conundrum of the emergence of cooperation, such as reward and punishment [9–15], persistence [16–18], social exclusion [19], reputation [20–24], voluntary participation [25], mobility [26–29], timescale diversity [30, 31], and conformism [32–35].

In many previous works, individuals were assumed to randomly select their learning neighbors. However, preferential selection is more realistic, so the effects of preferential selection on the evolution of cooperation have received much attention. Wu et al. [36] introduced a dynamic preferential selection mechanism into the evolutionary PDG, and proved that a simple selection rule can promote cooperation. Guan et al. then introduced a nonlinear attraction effect related to the payoff into the evolutionary PDG and considered two kinds of network structures: square lattices [37] and the regular small-world network [38]. Yang et al. [39] studied a degree-related preferential selection mechanism in evolutionary games. Shi et al. [40] found that payoff-based preferential selection can significantly enhance the emergence of public cooperation. Wang and Perc [41, 42] found that increasing the probability of selecting the most suitable neighbor can enhance cooperation. Wang et al. [43] proved that age-related preference selection favors the emergence of cooperation. Huang et al. [44] showed that individuals preferring to select neighbors who have a large degree of difference with themselves as a reference can significantly promote cooperation. Wu et al. [45] studied the influence of environment-based preferential selection on cooperation in evolutionary PDG, and showed that it can accelerate the formation of clusters to resist the invasion of defection. More recently, Wang et al. [46] introduced a preferential selection mechanism considering the satisfaction of payoffs and memory in the spatial PDG. Li et al. [47] showed that preferential selection based on dynamic reputation changes can facilitate cooperation in spatial multi-games.

So far, it has been assumed that individuals select role models using pure random selection or preferential selection, but the combined effects of the two selection mechanisms on cooperation have not been fully studied. In real life, people chooses the role model may neither completely according to a certain preferential selection rule nor according to a randomly selection rule. It is possible that some random factors are incorporated into the preferential selection process. In this paper, we study the transition towards preferential selection in the spatial PDG by introducing a normalized parameter *ε* to guide the system from pure random selection to preferential selection. We show that increasing *ε* (i.e., during this transition) has an effect on continuously enhancing the emergence and maintenance of cooperation.

The rest of this paper is organized as follows. We first specify the details of the evolutionary game model. We then report and analyze the simulation results. Finally, conclusions are provided in the last section.

## Model

In this paper, we consider the weak prisoner’s dilemma game on square lattices with periodic boundary conditions and complex networks. The population size is *N* = 10000 (for the square lattice, *L* = 100). Each individual occupies a node on the network and interacts with their direct neighbors. Individuals are initially designated either as a cooperator (C) or defector (D). Then, each individual *i* acquires the accumulated payoff *Π*_*i*_ through the pairwise interactions with their direct neighbors via the following rules: the reward for mutual cooperation is *R* = 1, the punishment for mutual defection is *P* = 0, and when two competing individuals have different strategies, the temptation to defect is *T* = *b* (1 ≤ *b* ≤ 2), and the payoff for the sucker is *S* = 0. Afterward, all the individuals select a nearest neighbor as the role model for strategy imitation. Specifically, individual *i* will select one direct neighbor *j* as a reference according to the probability *p*_*ij*_,

$$\begin{array}{c} p_{ij}=\varepsilon \frac{e^{\Pi_{j}}}{\sum_{z\in \Omega_{i}}e^{\Pi_{z}}}+\left(1-\varepsilon \right)\frac{1}{k_{i}} \end{array}$$ (1)

where Ω_*i*_ is the set of all direct neighbors of the individual *i*, and *k*_*i*_ is the degree of individual *i*. Here we introduce an adjustable parameter *ε* to characterize the strength of preferential selection. Obviously, when *ε* = 0, this selection scheme returns to the traditional version (random selection). For 0 < *ε* < 1, the combined effect of random selection and preferential selection is considered. *ε* = 1 corresponds to the situation in which the role model is selected purely according to the preferential selection rule.

After selecting the role model, all individuals update their strategies simultaneously according to the Fermi rule [48], i.e., the individual *i* adopts the strategy of the role model *j* with a probability determined by the Fermi function

$$\begin{array}{c} W\left(s_{i}\leftarrow s_{j}\right)=\frac{1}{1+\text{exp}[\left(\Pi_{i}-\Pi_{j}\right)/K]}, \end{array}$$ (2)

where *K* quantifies the amplitude of noise during the strategy imitation, with *K* = 0.1. Our simulations are performed by iterating the above procedure through over 10^4^ time steps, which is sufficient to ensure the system reaches a dynamical equilibrium state. To measure the impact of the combined effect of random selection and preferential selection on the evolution of cooperation, we monitor the cooperation level *F*_*C*_ and the data presented below are averaged over up to 50 realizations.

## Results

**Fig 1:**
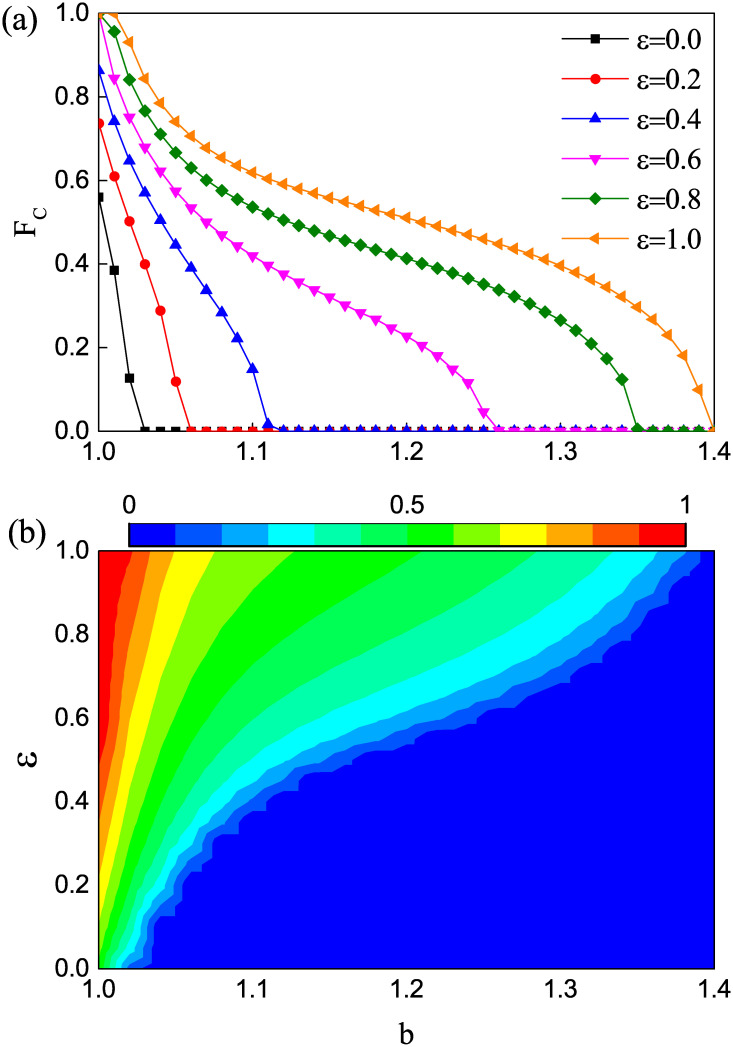
The effects of the strength of preferential selection *ε* and the temptation to defect *b* on the cooperation. (a) Fraction of cooperators *F*_*c*_ as a function of temptation to defect *b* with various values of the strength of preferential selection *ε*. (b) Contour plot for the equilibrium *F*_*c*_ on the *ε* − *b* parameter plane.

We first consider the effects of the strength of preferential selection *ε* and the temptation to defect *b* on the cooperation in evolutionary weak PDG on square lattices, as shown in Fig 1. Fig 1(a) shows the variation of the stable cooperation level *F*_*c*_ with *b* under different *ε*. Undoubtedly, no matter the value of *ε*, *F*_*c*_ gradually decreases with increasing temptation *b*. For *ε* = 0, the system returns to the traditional version, where the role model of each individual is chosen randomly from the neighbors, and the cooperation level sharply decreases to 0 with increasing temptation. For *ε* > 0, the preferential selection mechanism is introduced into the system. As *ε* increases, the individual becomes more likely to select a neighbor with a higher payoff to imitate. One can see that the introduction of the preferential selection mechanism can enhance the evolution of cooperation. To provide a holistic profile of the effects of *ε* and *b* on the evolution of cooperation, Fig 1(b) shows a contour plot encoding *F*_*c*_ on the *ε* − *b* parameter plane. As shown in this contour plot, the evolution of cooperation is influenced by the joint effects of *ε* and *b*. When *b* is fixed, *F*_*c*_ rises as we increase the strength of preferential selection *ε*. Moreover, a larger *ε* will lead to the need for a higher threshold of temptation *b* to eliminate cooperative behaviors. Obviously, this indicates that the preferential selection mechanism can tremendously benefit the emergence of cooperation in a population.

**Fig 2:**
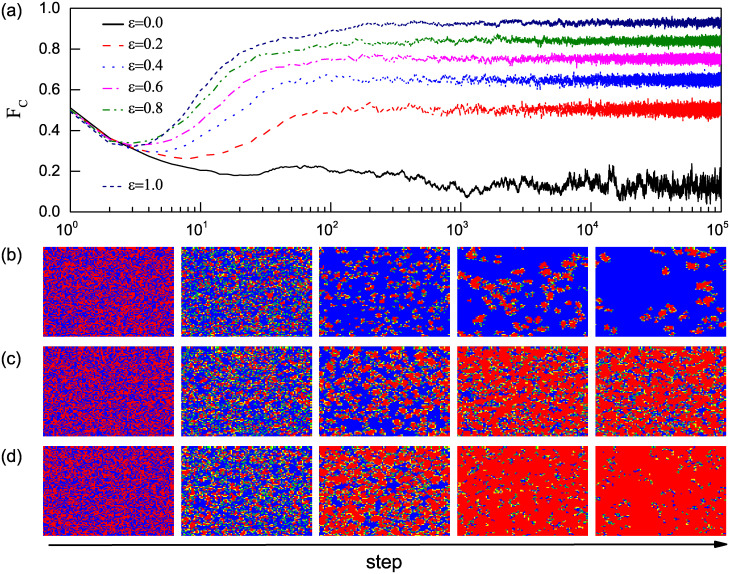
Time evolution of the fraction of cooperators and characteristic snapshots. (a) Time evolution of the fraction of cooperators *F*_*c*_ for different strengths of the preferential selection *ε*. (b)–(d) Characteristic snapshots of the distribution of the different individuals on square lattices. Red and yellow are cooperators and blue and green are defectors. More specifically, yellow denotes a cooperator whose strategy in the last round was defection, while green denotes a defector whose strategy in the last round was cooperation. From (b) to (d), the values of *ε* are 0.0, 0.5, and 1.0, respectively. The game parameter *b* is fixed to 1.02.

To investigate a potential mechanism for the enhancement of the emergence and maintenance of cooperation, we record in Fig 2(a) the time evolution of the fraction of cooperators *F*_*c*_ in the weak PDG on square lattices for six different strengths of preferential selection *ε* (*ε* = 0.0, 0.2, 0.4, 0.6, 0.8, 1.0). The temptation to defect *b* = 1.02. From Fig 2(a), one can see that the evolutionary process of the system is divided into two stages, the enduring (END) period and the expanding (EXP) period [49, 50]. In the END period, i.e., the early stage of the evolutionary process, *F*_*C*_ monotonically decreases no matter the value of *ε*, since the performance of the defectors is better than that of the cooperators. Moreover, one can observe that a larger *ε* results in a higher *F*_*C*_ and a shorter period of END. In the subsequent EXP period, the time series of *F*_*C*_ with different values of *ε* show two different trends. For *ε* > 0, *F*_*C*_ monotonically increases before reaching a plateau. However, for *ε* = 0 (the random selection version), *F*_*C*_ continues to decrease until reaching a dynamical equilibrium state.

Furthermore, we depict the characteristic snapshots of the distribution of different individuals in Fig 2(b)–2(d) to explore how the preferential selection mechanism influences the evolution of cooperation. Initially, all individuals are randomly distributed on the square lattice. In the early stage of the game (i.e., the END period), the snapshots corresponding to different *ε* do not show much difference. Many boundary and isolated cooperators are changed into defectors as they are exploited by the surrounding defectors (see the second column snapshots). However, the difference between snapshots with *ε* = 0 and *ε* > 0 becomes remarkable in the following EXP stage. Compared with the traditional model (*ε* = 0), the introduction of preferential selection (*ε* > 0) can significantly boost the expansion of cooperative clusters. For *ε* > 0, the neighbor with the highest payoff is more likely to be chosen as the role model of a boundary individual. At the beginning of the EXP stage, some small cooperative clusters have been formed, in which the internal cooperators have advantages in payoffs. The boundary individuals are then more inclined to select internal cooperators to imitate. As shown in Fig 2(b)–2(d), the larger *ε*, the more large and compact the cooperative clusters.

**Fig 3:**
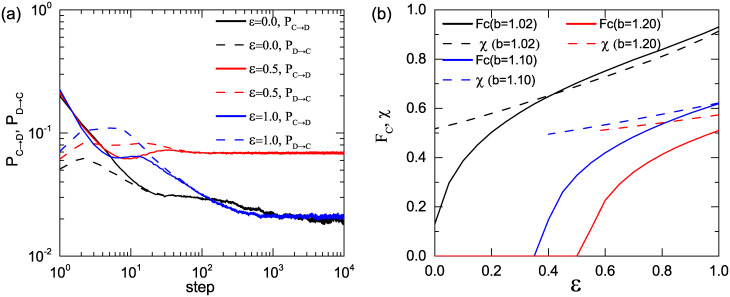
The analysis of strategies transform and the compactness of cooperator clusters. (a) Time evolution of the fraction of individuals whose strategies transform from cooperation to defection (defection to cooperation) *P*_*C*→*D*_ (*P*_*D*→*C*_) for *ε* = 0.0, 0.5, 1.0. The game parameter *b* = 1.02. (b) Fraction of cooperators *F*_*C*_ (solid line) and ratio *χ* (dashed line) as functions of the strength of preferential selection *ε* for *b* = 1.02, 1.10, 1.20.

To further support the above explanation, we present the time evolution of the fraction of individuals whose strategies transform from cooperation to defection (defection to cooperation) *P*_*C*→*D*_ (*P*_*D*→*C*_) for different *ε* in Fig 3(a). At the END stage, one can obviously see that *P*_*C* → *D*_ is always higher than *P*_*D*→*C*_ for any given value of *ε*, so the cooperators cannot defend against the invasion of the defectors. While it is the reverse for *ε* = 0.5 and 1.0 when the system is entering into the EXP period, the introduction of preferential selection can facilitate the spreading of cooperation. Moreover, the effect of the strength of preferential selection on the evolution of cooperation is further investigated. We introduce the quantity *χ* = *ρ*_*CC*_/*ρ*_*CL*_ to quantify the compactness of cooperator clusters, where *ρ*_*CC*_ is the fraction of cooperator-cooperator links (i.e., links connect two cooperators), and *ρ*_*CL*_ denotes the ratio of direct links ending at a cooperator. Clearly, a larger *χ* means the cooperative clusters in the population are more compact, and *χ* = 1 means the full cooperation state has been reached. Fig 3(b) shows the equilibrium *F*_*C*_ (solid line) and quantity *χ* (dashed line) as functions of *ε* for *b* = 1.02, 1.10, 1.20. For each given *b*, the equilibrium *F*_*C*_ and *χ* increase monotonically increasing *ε*, which indicates that the introduction of preferential selection can facilitate and stabilize the formation of compact cooperative clusters, and the cooperators can more easily avoid the invasion of defectors.

**Fig 4:**
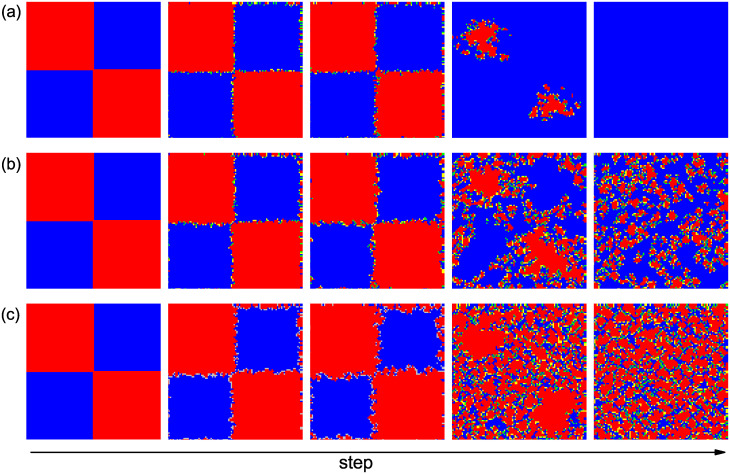
Characteristic snapshots of the distribution of different individuals on square lattices with a prepared initial condition. Red and yellow represent cooperators and blue and green represent defectors. More specifically, yellow denotes a cooperator whose strategy in the last round was defection, while green marks a defector whose strategy in the last round was cooperation. The values of *ε* are set to be 0 (top row), 0.5 (middle row), 1 (bottom row) and *t* = 1, *t* = 10, *t* = 50, *t* = 100, *t* = 10000 from left to right. The game parameter *b* is fixed to 1.1.

To better determine why the preferential selection mechanism can more effectively stabilize the formation of cooperative clusters than pure random selection, we depict three representative spatial evolutionary snapshots on square lattices in Fig 4, in which the simulation is started from a prepared initial condition. In the absence of preferential selection (*ε* = 0.0), the initial compact cooperative clusters are not able to defend against the invasion of defection, and eventually, the cooperative behavior in the population goes extinct. However, the cooperative clusters are strong when the preferential selection scheme is introduced, and the introduction of preferential selection can stabilize the coexistence of cooperation and defection. For *ε* = 1.0, the cooperative clusters can even outperform the defectors, and cooperation becomes the dominant strategy in the population even if the temptation *b* is not small.

**Fig 5:**
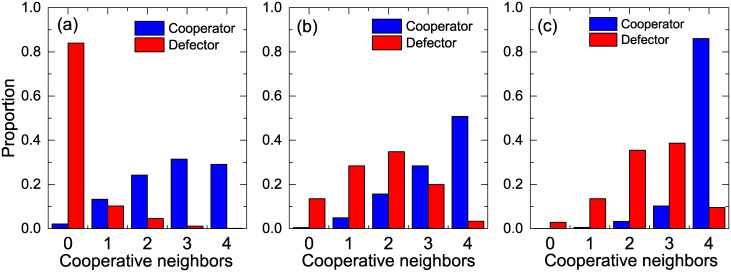
Distribution of cooperative neighbors of focal cooperators and focal defectors. Results of focal cooperators and focal defectors are denoted by blue and red columns, respectively. From (a) to (c), the values of *ε* are 0.0, 0.5, and 1.0, respectively. The game parameter *b* is fixed to 1.02.

In addition, we studied the distribution of cooperative neighbors of the focal cooperators and focal defectors in Fig 5. As shown in Fig 5(a), for *ε* = 0.0, cooperative neighbors can hardly survive around the focal defectors. This is because defection is more advantageous, and cooperators can only form compact clusters to resist the invasion of defectors. For *ε* = 0.5, about half of the focal cooperators are completely surrounded by cooperative neighbors, indicating that the introduction of preferential selection can facilitate the formation of compact cooperative clusters. For *ε* = 1.0, the proportion of focal cooperators with four cooperative neighbors is even more than 0.8, again confirming our conclusion.

**Fig 6:**
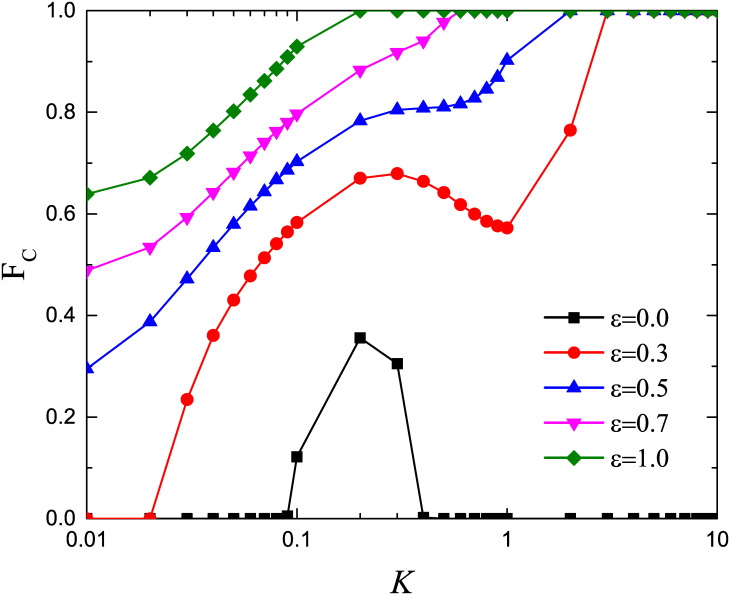
Effect of the amplitude of noise *K* on the fraction of cooperators *F*_*C*_ for different strengths of the preferential selection *ε*. The game parameter *b* is fixed to 1.02.

It is significant to explore the influence of noise *K* on the level of cooperation. The above results are obtained when *K* = 0.1. Fig 6 shows that qualitative features of the results remains unchanged under different noise *K*, that is, the proportion of cooperators increases with the strength of preferential selection. In addition, for *ε* = 0.0, we can observe that there exist an optimal *K* value that enables the cooperator to survive, which is consistent with previous work. For *ε* > 0.0, the increase of noise *K* is conducive to promoting cooperation.

**Fig 7:**
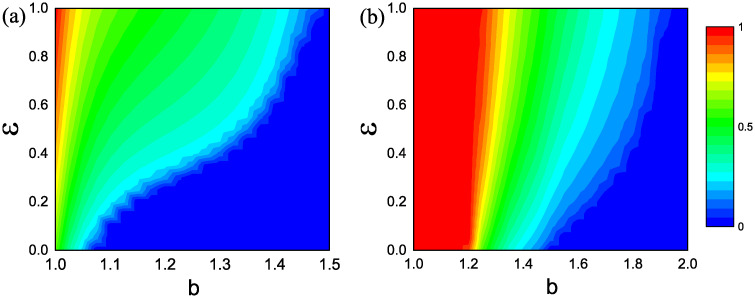
Contour plots for the fraction of cooperators *F*_*C*_ on the *ε* − *b* parameter plane for different topologies with the average degree 〈*k*〉 = 4. (a) is for random regular networks and (b) is for ER networks. Simulations are carried out for the network size *N* = 10000.

Finally, we have also explored the robustness of the above results for other kinds of topological structures. Fig 7 shows contour plots encoding the cooperation level *F*_*C*_ on the *ε* − *b* parameter plane, and the simulation results in Fig 7(a) and 7(b) are carried out on random regular networks (RRNs) [51] and Erdös-Rényi (ER) random [52] networks with the same average degree 〈*k*〉 = 4. From Fig 7, we can observe that the steady cooperation level *F*_*C*_ increases with the strength of the preferential selection *ε*, regardless of whether the underlying interaction network is an RRN or ER network, and the introduction of the preferential selection mechanism can enhance the emergence of cooperation in social dilemmas. This is also consistent with the numerical results shown in Fig 1(b), which are carried out on square lattices, except that higher equilibrium *F*_*C*_ could be obtained on RRN and ER networks due to stronger network reciprocity. These results indicate that the preferential selection mechanism facilitates the evolution of cooperation significantly regardless of the underlying topological structure.

## Conclusion

In this paper, we have investigated the effects of the selection scheme of role models on cooperation in spatial evolutionary PDGs. We have proposed a weak PDG model by combining pure random selection and preferential selection rules. Specifically, we have introduced a normalized parameter *ε* to control the weights of these two types of selection rules. We have focused on the transition towards preferential selection in the spatial PDG by controlling the tunable parameter *ε* to guide the system from pure random selection to preferential selection. Through numerical simulations, we found that the introduction of a preferential selection mechanism can significantly alleviate social dilemmas and enhance the effect of network reciprocity, which can effectively enhance the emergence and maintenance of cooperative behaviors among selfish individuals. The critical threshold of the temptation *b* for the annihilation of cooperative behaviors becomes larger as *ε* increases, which means that the stronger the preferential selection, the more conducive it is to the evolution of cooperation. Moreover, we have provided some detailed explanations for the above simulation results from the perspective of the fraction of strategy transition and the nature of the cooperative clusters. Finally, we have discussed the robustness of the results for noise *K* and different topologies. We believe our study takes the understanding of preferential selection in spatial evolutionary games one step further.

## References

[1] Colman AM. Game theory and its applications: In the social and biological sciences. Psychology Press; 2013.

[2] Nowak MA. Evolutionary dynamics: exploring the equations of life. Harvard University Press; 2006.

[3] Axelrod R, Hamilton WD. The evolution of cooperation. Science. 1981; 211(4489):1390–1396. doi: 10.1126/science.7466396 7466396

[4] Szabó G, Fáth G. Evolutionary games on graphs. Physics Reports. 2007; 446(4-6):97–216. doi: 10.1016/j.physrep.2007.04.004

[5] Wang Z, Bauch CT, Bhattacharyya S, et al. Statistical physics of vaccination. Physics Reports. 2016; 664:1–113. doi: 10.1016/j.physrep.2016.06.004

[6] Perc M, Jordan JJ, Rand DG, et al. Statistical physics of human cooperation. Physics Reports. 2017; 687:1–51. doi: 10.1016/j.physrep.2017.05.004

[7] Wang Z, Li R, Jin X, et al. Emergence of Social Norms in Metanorms Game With High-Order Interaction Topology. IEEE Transactions on Computational Social Systems. 2022; 1–16. doi: 10.1109/TCSS.2022.3204177

[8] Nowak MA, May RM. Evolutionary games and spatial chaos. Nature. 1992; 359(6398):826–829. doi: 10.1038/359826a0

[9] Perc M, Szolnoki A. Self-organization of punishment in structured populations. New Journal of Physics. 2012; 14(4):043013. doi: 10.1088/1367-2630/14/4/043013

[10] Wang Z, Szolnoki A, Perc M. Rewarding evolutionary fitness with links between populations promotes cooperation. Journal of Theoretical Biology. 2014; 349:50–56. doi: 10.1016/j.jtbi.2014.01.037 24508726

[11] Yang H, Wu Z, Rong Z, et al. Peer pressure: Enhancement of cooperation through mutual punishment. Physical Review E. 2015; 91(2):022121. doi: 10.1103/PhysRevE.91.022121 25768472

[12] Wang X, Nie S, Jiang L, et al. Role of delay-based reward in the spatial cooperation. Physica A: Statistical Mechanics and Its Applications. 2017; 465:153–158. doi: 10.1016/j.physa.2016.08.014

[13] Han D, Yan S, Li D. The evolutionary public goods game model with punishment mechanism in an activity-driven network. Chaos, Solitons & Fractals. 2019; 123:254–259. doi: 10.1016/j.chaos.2019.04.015

[14] Liu L, Chen X, Szolnoki A. Evolutionary dynamics of cooperation in a population with probabilistic corrupt enforcers and violators. Mathematical Models and Methods in Applied Sciences. 2019; 29(11):2127–2149. doi: 10.1142/S0218202519500428

[15] Liu L, Chen X. Effects of interconnections among corruption, institutional punishment, and economic factors on the evolution of cooperation. Applied Mathematics and Computation. 2022; 425:127069. doi: 10.1016/j.amc.2022.127069

[16] Huang C, Dai Q. Persistence paves the way for cooperation in evolutionary games. EPL(Europhysics Letters). 2017; 118(2):28002. doi: 10.1209/0295-5075/118/28002

[17] Huang C, Dai Q, Li H. Leaders should be more persistent in evolutionary social dilemmas. EPL(Europhysics Letters). 2018; 124(1):18001. doi: 10.1209/0295-5075/124/18001

[18] Zhang L, Huang C, Li H, et al. Aspiration-dependent strategy persistence promotes cooperation in spatial prisoner’s dilemma game. EPL(Europhysics Letters). 2019; 126(1):18001. doi: 10.1209/0295-5075/126/18001

[19] Liu L, Xiao Z, Chen X, et al. Early exclusion leads to cyclical cooperation in repeated group interactions. Journal of the Royal Society Interface. 2022; 19(188):20210755. doi: 10.1098/rsif.2021.0755 35317651 PMC8941418

[20] Wang Z, Wang L, Yin Z, et al. Inferring reputation promotes the evolution of cooperation in spatial social dilemma games. PloS one. 2012; 7(7):e40218. doi: 10.1371/journal.pone.0040218 22808120 PMC3392274

[21] Yang H, Wang Z. Promoting cooperation by reputation-driven group formation. Journal of Statistical Mechanics: Theory and Experiment. 2017; 2017(2):023403. doi: 10.1088/1742-5468/aa569f

[22] Xia C, Gracia-Lázaro C, Moreno Y. Effect of memory, intolerance, and second-order reputation on cooperation. Chaos: An Interdisciplinary Journal of Nonlinear Science. 2020; 30(6):063122. doi: 10.1063/5.0009758 32611098

[23] Zhang L, Zhang L, Huang C. Defectors in bad circumstances possessing higher reputation can promote cooperation. Chaos: An Interdisciplinary Journal of Nonlinear Science. 2022; 32(4):043114. doi: 10.1063/1.2784384 35489841

[24] Xia C, Hu Z, Zhao D. Costly reputation building still promotes the collective trust within the networked population. New Journal of Physics. 2022; 24(8):083041. doi: 10.1088/1367-2630/ac8898

[25] Szabó G, Hauert C. Phase transitions and volunteering in spatial public goods games. Physical Review Letters. 2002; 89(11):118101. doi: 10.1103/PhysRevLett.89.118101 12225171

[26] Cardinot M, ORiordan C, Griffith J, et al. Mobility restores the mechanism which supports cooperation in the voluntary prisoners dilemma game. New Journal of Physics. 2019; 21(7):073038. doi: 10.1088/1367-2630/ab3064

[27] Xiao Z, Chen X, Szolnoki A. Leaving bads provides better outcome than approaching goods in a social dilemma. New Journal of Physics. 2020; 22:023012. doi: 10.1088/1367-2630/ab6a3b

[28] Zhang L, Huang C, Li H, et al. Effects of directional migration for pursuit of profitable circumstances in evolutionary games. Chaos, Solitons & Fractals. 2021; 144:110709. doi: 10.1016/j.chaos.2021.110709

[29] Zhang L, Huang C, Li H, et al. Migration based on historical payoffs promotes cooperation in continuous two-dimensional space. EPL(Europhysics Letters). 2021; 134(6):68001. doi: 10.1209/0295-5075/134/68001

[30] Rong Z, Wu Z, Hao D, et al. Diversity of timescale promotes the maintenance of extortioners in a spatial prisoner’s dilemma game. New Journal of Physics. 2015; 17(3):033032. doi: 10.1088/1367-2630/17/3/033032

[31] Xu X, Rong Z, Tian Z, et al. Timescale diversity facilitates the emergence of cooperation-extortion alliances in networked systems. Neurocomputing. 2019; 350:195–201. doi: 10.1016/j.neucom.2019.06.096

[32] Szolnoki A, Perc M. Conformity enhances network reciprocity in evolutionary social dilemmas. Journal of The Royal Society Interface. 2015; 12(103):20141299. doi: 10.1098/rsif.2014.1299 25540242 PMC4305429

[33] Yang H, Tian L. Enhancement of cooperation through conformity-driven reproductive ability. Chaos, Solitons & Fractals. 2017; 103:159–162. doi: 10.1016/j.chaos.2017.06.005

[34] Liu X, Huang C, Dai Q, et al. The effects of the conformity threshold on cooperation in spatial prisoner’s dilemma games. EPL(Europhysics Letters). 2019; 128(1):18001. doi: 10.1209/0295-5075/128/18001

[35] Lin J, Huang C, Dai Q, et al. Evolutionary game dynamics of combining the payoff-driven and conformity-driven update rules. Chaos, Solitons & Fractals. 2020; 140:110146. doi: 10.1016/j.chaos.2020.110146

[36] Wu Z, Xu X, Huang Z, et al. Evolutionary prisoners dilemma game with dynamic preferential selection. Physical Review E. 2006; 74(2):021107. doi: 10.1103/PhysRevE.74.021107 17025393

[37] Guan J, Wu Z, Huang Z, et al. Promotion of cooperation induced by nonlinear attractive effect in spatial Prisoner’s Dilemma game. EPL(Europhysics Letters). 2006; 76(6):1214. doi: 10.1209/epl/i2006-10381-4

[38] Guan J, Wu Z, Huang Z, et al. Prisoner’s dilemma game with nonlinear attractive effect on regular small-world networks. Chinese Physics Letters. 2006; 23(10):2874. doi: 10.1088/0256-307X/23/10/068

[39] Yang HX, Wang WX, Wu ZX, et al. Diversity-optimized cooperation on complex networks. Physical Review E. 2009; 79(5):056107. doi: 10.1103/PhysRevE.79.056107 19518521

[40] Shi DM, Yang HX, Hu MB, et al. Preferential selection promotes cooperation in a spatial public goods game. Physica A: Statistical Mechanics and its Applications. 2009; 388(21):4646–4650. doi: 10.1016/j.physa.2009.07.031

[41] Wang Z, Perc M. Aspiring to the fittest and promotion of cooperation in the prisoner’s dilemma game. Physical Review E. 2010; 82(2):021115. doi: 10.1103/PhysRevE.82.021115 20866783

[42] Perc M, Wang Z. Heterogeneous aspirations promote cooperation in the prisoner’s dilemma game. PLOS one. 2010; 5(12):e15117. doi: 10.1371/journal.pone.0015117 21151898 PMC2997779

[43] Wang Z, Wang Z, Yang YH, et al. Age-related preferential selection can promote cooperation in the prisoner’s dilemma game. International Journal of Modern Physics C. 2012; 23(02):1250013. doi: 10.1142/S0129183112500131

[44] Huang C, Dai Q, Cheng H, et al. Preferential selection based on degree difference in the spatial prisoner’s dilemma games. EPL(Europhysics Letters). 2017; 120(1):18001. doi: 10.1209/0295-5075/120/18001

[45] Wu Y, Zhang S, Zhang Z. Environment-based preference selection promotes cooperation in spatial prisoner’s dilemma game. Scientific Reports. 2018; 8(1):1–9. doi: 10.1038/s41598-018-34116-0 30353150 PMC6199282

[46] Wang B, Kang W, Sheng J, et al. Preferential selection based on payoff satisfaction and memory promotes cooperation in the spatial prisoner’s dilemma games. EPL(Europhysics Letters). 2020; 129(3):38002. doi: 10.1209/0295-5075/129/38002

[47] Li X, Hao G, Wang H, et al. Reputation preferences resolve social dilemmas in spatial multigames. Journal of Statistical Mechanics: Theory and Experiment. 2021; 2021(1):013403. doi: 10.1088/1742-5468/abd4cf

[48] Szabó G, Tőke C. Evolutionary prisoner’s dilemma game on a square lattice. Physical Review E. 1998; 58(1):69. doi: 10.1103/PhysRevE.58.69

[49] Wang Z, Kokubo S, Tanimoto J, et al. Insight into the so-called spatial reciprocity. Physical Review E. 2013; 88(4):042145. doi: 10.1103/PhysRevE.88.042145 24229153

[50] Tanimoto J. Impact of deterministic and stochastic updates on network reciprocity in the prisoner’s dilemma game. Physical Review E. 2014; 90(2):022105. doi: 10.1103/PhysRevE.90.022105 25215687

[51] Wormald NC. Models of random regular graphs. London Mathematical Society Lecture Note Series. 1999; 239-298.

[52] Erdös P, Rényi A. On the evolution of random graphs. Publication of the Mathematical Institute of the Hungarian Academy of Sciences. 1960; 5(1):17–60.

